# Anti-inflammatory effects of 1,8-cineol via NF-κB/COX-2 pathway in BEAS-2B cells and alleviates bronchoconstriction and airway hyperreactivity in ovalbumin sensitized mice

**DOI:** 10.3389/fimmu.2026.1714915

**Published:** 2026-03-27

**Authors:** Yanhong Wang, Jiaming Zhu, Xu Zhang, Fei Tong, Qiaoping Xu

**Affiliations:** 1 Department of Pharmacy, Second Affiliated Hospital, School of Medicine, Zhejiang University, Hangzhou, China; 2 Fourth Clinical Medical College of Zhejiang Chinese Medical University, Affiliated Hangzhou First People’s Hospital, Hangzhou, China; 3 Department of Pharmacy, Zhujiang Hospital, Southern Medical University, Guangzhou, Guangdong, China; 4 Department of Clinical Pharmacology, Key Laboratory of Clinical Cancer Pharmacology and Toxicology Research of Zhejiang Province, Affiliated Hangzhou First People’s Hospital, Cancer Center, Westlake University, Hangzhou, China

**Keywords:** 1,8-cineol, airway inflammation, airway remodeling, NF-κB/COX-2 pathway, ovalbumin-induced acute asthma, Th2 cytokine

## Abstract

**Objective:**

Asthma is a chronic inflammatory airway disease characterized by airway remodeling and hyperresponsiveness, driven in part by TGF-β1-induced epithelial-mesenchymal transition (EMT). The natural compound 1,8-cineol, derived from *Eucalyptus globulus*, has shown anti-inflammatory potential. This study aimed to investigate its protective effects against EMT and airway inflammation via the NF-κB/COX-2 pathway.

**Methods:**

*In vivo*, ovalbumin-sensitized BALB/c mice were treated with 1,8-cineol (50 mg/kg) to evaluate airway resistance, lung compliance, and inflammatory markers (IgE, IL-4, IL-13, IL-17). Histopathological changes were assessed via H&E and PAS staining. *In vitro*, TGF-β1-stimulated BEAS-2B cells were treated with 1,8-cineol to analyze EMT markers (α-SMA, E-cadherin, N-cadherin), migration capacity, and NF-κB/COX-2 signaling using RT-qPCR, Western blotting, and transwell assays.

**Results:**

1,8-cineol significantly attenuated airway hyperresponsiveness and reduced EMT markers (α-SMA, N-cadherin) in OVA-sensitized mice, while improving lung compliance. In BEAS-2B cells, it suppressed TGF-β1-induced EMT and migration without cytotoxicity. Mechanistically, 1,8-cineol downregulated NF-κB phosphorylation and COX-2 expression. OVA challenge elevated serum IgE and BALF cytokines (IL-4, IL-13, IL-17), which were mitigated by 1,8-cineol.

**Conclusions:**

1,8-cineol inhibits TGF-β1-driven EMT and airway inflammation by modulating the NF-κB/COX-2 pathway, highlighting its therapeutic potential for asthma.

## 1 Introduction

Asthma is a heterogeneous chronic inflammatory disease affecting airways in the lung, which is closely associated with airway inflammation and airway remodeling, and characterized by the airway hyperresponsiveness (AHR) (1). According to the 2015 Global Burden of Disease Report, asthma affects an estimated 300 million people worldwide (2). Chronic inflammation is often accompanied by airway remodeling in asthma. Although the pathogenesis of airway remodeling remains elusive, the chronic inflammatory response in the airway is a major force driving the remodeling process. Invasive inflammatory cells, primarily eosinophils and mast cells, release overactive enzymes such as major basic proteins (MBP),neurotoxins, peroxidase, and cationic proteins that cause lung tissue damage in asthmatic patients, triggering abnormalities the usual repair route (3). Therefore, for recurrent chronic asthma, treatment options should focus on how to effectively slow airway remodeling. The combination of inhaled corticosteroids (ICS) and long acting beta-agonists (LABA) can relieve asthma symptoms and reduce the frequency of wheezing attacks. But for those with asthma who fail to gain control through ICS and LABA, treatment options are limited. There is usually no additional benefit associated with an increased dose of ICS, but there is an increased risk of side effects.

In the pathogenesis of severe asthma, not only eosinophils but also mast cells or neutrophils play important roles. Airway wall remodeling has been described as changes to the composition, thickness, and volume of the airway wall (4). EMT is a critical process in which epithelial cells lose polarity and acquire mesenchymal characteristics, contributing to airway remodeling in asthma. As reported, The EMT process can induce organ fibrosis, tissue repair and cancer metastasis. Suppression of EMT is demonstrated to alleviate airway hyperresponsiveness and fibrosis. TGF-β1 is a well-known cytokine that contributes to EMT and collagen deposition which can exacerbate the development of asthma (5). Also, bone marrow-derived mesenchymal stem cells reduce EMT in the airway epithelium, thereby alleviating allergic inflammation in the airways and decreasing airway wall remodeling in asthmatic rats (6). Thus, EMT has been proposed as a promising target for the treatment of asthma.

1,8-Cineol (eucalyptol), the predominant monoterpene in eucalyptus essential oil, has been historically employed in traditional medicine for respiratory ailments. Modern pharmacological studies have confirmed its broad anti-inflammatory and antioxidant properties, with potential therapeutic effects in peptic ulcer disease, neurological disorders, cardiovascular diseases, and even cancer (7). Notably, Qiu et al. study demonstrated that enteric capsules combining 1,8-cineole, limonene, and α-pinene significantly attenuated airway inflammation and mucus hypersecretion in LPS-induced chronic bronchitis models by suppressing TLR4/NF-κB signaling-reducing key cytokines including TNF-α and IL-6, mucins and phospho-p65 expression in bronchial epithelium (8). However, its therapeutic potential in modulating EMT through the NF-κB/COX-2 signaling axis in allergic asthma remains to be explored.

Clinical studies have demonstrated that 1,8-cineole exhibits steroid-like anti-inflammatory effects by inhibiting arachidonic acid metabolism and cytokine production. A double-blind, placebo-controlled trial offered robust clinical evidence supporting the anti-inflammatory efficacy of 1,8-cineole in asthma management. The trial involved 32 patients with severe asthma and significant glucocorticoid dependence. Patients treated with 1,8-cineole experienced an average 36% reduction in oral glucocorticoid dosage (approximately 3.75 mg/day), compared to a 7% reduction (approximately 0.91 mg/day) in the placebo group. Furthermore, those receiving 1,8-cineole maintained stable lung function and had significantly lower cumulative glucocorticoid exposure than the placebo group. Notably, the study also confirmed the favorable safety profile of 1,8-cineole, highlighting its potential for long-term therapeutic use in asthma and chronic obstructive pulmonary disease (COPD). These findings underscore the potential of 1,8-cineole as an effective anti-inflammatory agent for treating airway diseases (9).

The main pathological changes of airway remodeling were epithelial injury and plasticity, fibroblast/myofibroblast increase, airway smooth muscle hyperplasia/hypertrophy, basement membrane thickening and angiogenesis. Airway remodeling is often associated with a severe phenotype of the disease. Chronic allergic inflammation is the initiation time of remodeling as well as the above pathological changes. IL-4 regulates the recruitment of eosinophils, the class-switching of immunoglobulins and Th2 differentiation, while IL-13 directly regulates structural cells, including fibroblasts and epithelial cells, promoting excessive mucus secretion and extracellular matrix (ECM) deposition. Although the signaling pathways are the same, IL-13 becomes the dominant cytokine in the remodeling process, including excessive mucus secretion, fibrosis and smooth muscle hypertrophy. IL-4 mainly amplifies the inflammatory cascade by driving IgE switching, promotes Th2 cell polarization to maintain cytokine release, and induces the recruitment of eosinophils by chemokines. Chronic allergic inflammation induces these changes, which are particularly pronounced in transgenic mice overexpressing IL-4, IL-5, and IL-13. These mice show goblet cell hyperplasia, mucus accumulation, thickening of the airway basement membrane, and increased airway reactivity. Elevated levels of IL-4, IL-5, and IL-13 have been detected in the bronchial tissue and BALF of asthmatic patients, highlighting these cytokines as critical targets for asthma therapy (10). In addition, IL-4 and IL-13 are also very crucial in airway dysfunction and reconstruction following chronic exposure to allergen (11).

NF-kappa B is closely associated with rheumatoid arthritis, atherosclerosis, multiple sclerosis, chronic inflammatory demyelinating poly radiculoneuritis, inflammatory bowel disease and asthma (12). It plays a role in inflammatory reactions through the expression of inflammatory media, adhesion molecules, and enzymes (13). Inhibition of NF-kappa B expression may reduce airway inflammation and airway modification (14). Bay-11–7082 is currently widely used as an anti-inflammatory agent and is also marketed as an inhibitor of transcription factor NF-kappa B activation (15). In the ground state, NF-kappa B binds to a regulatory protein called inhibitors of κB and remains inactive in the cell brush. In response to inflammatory cytokines or cellular stresses, IκB is phosphorylated by IκB kinase (IKK) along with IκB ubiquitination and proteolysis degradation of IκB, leading to kapa B activation and nuclear transfer (16). Bay-11–7082 has been reported to inhibit IKK activity and to maintain the inactive complex of NF-κB with IκB.

NF-κB signaling pathway also plays a key role in regulating inflammatory response through COX transcription (17). iNOS and COX-2 induced by nuclear transcription factor κB are important mediators in the occurrence of pulmonary inflammation. In addition, the expression of iNOS and the activation of NF-κB by COX-2 increased (18, 19). iNOS and COX-2 themselves, on the other hand, are involved in the activation of NF-κB, which can subsequently induce other inflammatory mediators and cells (20). In addition, studies have shown that IL-4 can modify the activation of nuclear factor κB and other factor-induced NF-κB (21), and NF-κB also plays an important role in the pathogenesis of IL-13-induced asthma tissue damage (22). Activation of NF-κB is critical in inflammatory responses by inducing transcription of pro-inflammatory genes and has been associated with airway inflammatory diseases such as asthma (16). As the downstream of NF-κB, COX-2 has long been considered a protein that plays a major role in airway inflammation in asthma and is highly induced by a variety of stimuli such as cytokines and oxidative stress (23), and its expression has been shown to be significantly elevated in asthmatic patients and mice compared to normal subjects (24). The protective effect of natural products in asthma models by modulating the expression of iNOS and COX-2 is well established. The increase of COX-2 expression can lead to the increase of prostaglandin E2 (PGE2), the main COX-2-induced product, leading to airway smooth muscle contraction. Studies have shown that NF-κB-induced COX-2 is an important mediator of pulmonary inflammation, and the activation of NF-κB can also promote the expression of COX-2. On the other hand, COX-2 itself is also involved in the activation process of NF-κB, leading to induction of other inflammatory mediators and cells (19). This is also the first time in this paper that it has been discovered that 1,8-cineol has an effect on the epithelial-mesenchymal transition in asthma.

## 2 Materials and methods

### 2.1 Ethical approval of the study protocol

The study protocol was approved by the Animal Care and Use Committee of Zhejiang Chinese Medical University. The researchers were unaware during the process of histological scoring and quantitative analysis.

### 2.2 Chemicals and reagents

1,8-Cineol was used from Soledum^®^ capsules (Klosterfrau Healthcare Group, Cassellamed GmbH & Co. KG, Cologne, Germany). Native extract (0.6 mg/μl, 600 mg/ml, 1,8-Cineol) was stored at 4°C, while stock solution was prepared by solving native extract in ethanol (100mg/ml) followed by final diluting with DMEM High Glucose (Biochrom, Berlin, Germany; 1mg/ml).

To identify BAY-11–7082 dose, we carried out preliminary experiments with different doses (5, 10 and 20 mg/kg) of the compound, titrated against the histological damage. The dose of 20 mg/kg was the most effective and therefore we used this dosage in the study to keep to the minimum, for ethical issues, the number of experimental animals. This dose was similar to that used in a previously published paper investigating the effects of BAY-11–7082 on depressed wound healing in diabetic animals (16). Since asthma is a systemic disease we chose the intraperitoneal route of administration.

### 2.3 Animals

Specific pathogen-free female BALB/c mice (6 weeks old) were purchased from the Experimental Animals Center of Zhejiang University of Traditional Chinese Medicine. Before experimentation, all mice were maintained under standard conditions, and survived on distilled water and standard chow adlibitum, for 7 days. Animal procedures were approved by the ZheJiang University Animal Care and Use Committee and performed in accordance with established animal care, use, and experimental guidelines.

### 2.4 Asthma model establishment

**Figure 1:**
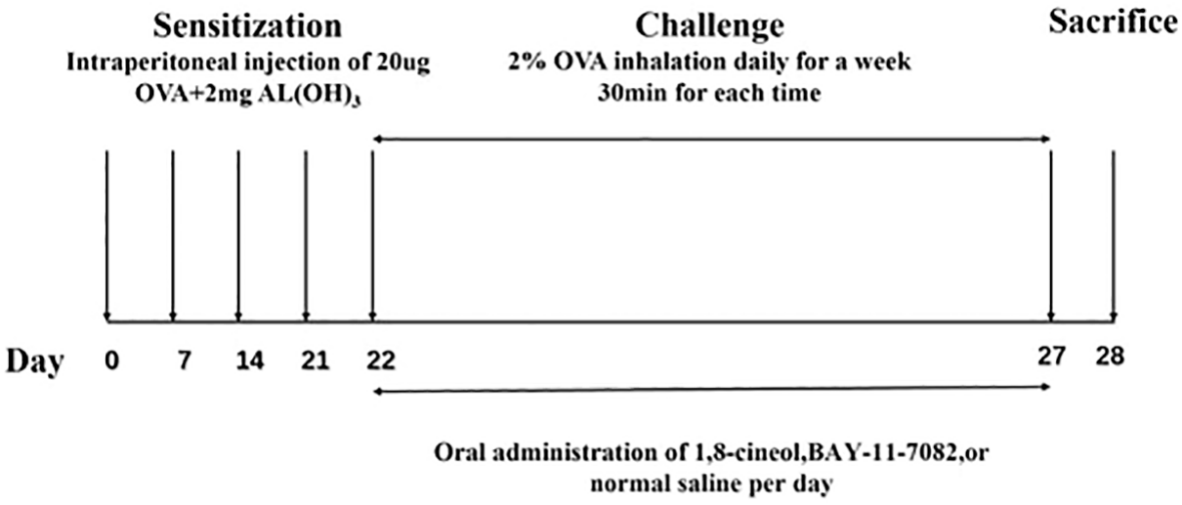
Experimental protocol for ovalbumin (25)-induced asthma model.

Thirty Mice were randomly allocated into groups of six mice each: normal control group (Control, n=6); OVA-induced asthma model group (OVA, n=6), asthma model combined with 1,8-cineol treatment group[OVA + 1,8-cineol (50 mg/kg),n=6], OVA +BAY-11-7082(20mg/kg) and OVA+BAY-11-7082 (20mg/kg)+1,8-cineol(50 mg/kg), n=6 in each group. To establish a model of allergic asthma, mice were sensitized and challenged with ovalbumin (25) by intraperitoneal injection and atomization inhalation as mentioned previously (26). An OVA-induced mice model of allergic asthma was established by intraperitoneally injection of 20 μg OVA (Grade V; Sigma-Aldrich, St. Louis, MO) and 2 mg aluminum hydroxide (Thermo Scientific) at day 0, 7, 14 and 21 (Sensitization stage). Non-OVA-challenged mice were sensitized and challenged with 0.9% saline alone. From day 22 (Challenge stage), mice were given 2% OVA ultrasonic atomization inhalation for 30 min once a day for consecutive 7 days. Each time of challenge, mice in the 1,8-cineol and BAY-11–7082 groups were gavaged with 50 mg/kg and 20 mg/kg (4, 27) (**Figure 1**).

### 2.5 Bronchoalveolar lavage fluid collection

BALF was collected by lavage of the lung twice with 0.5 ml of cold physiologic saline via a tracheal catheter. BALF was centrifuged immediately (2,000 g, 5 min, 4°C). The supernatant was used for cytokine measurements. The cell pellet was resuspended in 0.5 ml of phosphate-buffered saline (PBS) and used for total and differential cell counts, which were measured by H&E-stained cytocentrifuges.

### 2.6 Measurement of cytokine levels in BALF and cell supernatants

Concentrations of IL-4、IL-13 and IL-17 in BALF were measured by ELISAs according to manufacturer (R&D Systems) instructions.

### 2.7 Real-time reverse transcription-quantitative PCR

Total RNA was isolated from lung tissues by TRIzol^®^ Reagent according to manufacturer (Invitrogen, Carlsbad, CA, United States) instructions. Reverse transcription was carried out with the PrimeScript^®^ RT Reagent Kit (TaKaRa Biotechnology, Shiga, Japan). Next, RT-qPCR was done with the SYBR^®^ Green Premix Ex Taq kit (TaKaRa Biotechnology) on a LightCycler^®^ 480 system (Roche, Basel, Switzerland). mRNA expression of target genes was normalized to GAPDH expression in the same sample. The primers we used (forward and reverse, respectively)were: 5′ - TGTGGGCATCAATGGATTTGG - 3′ and 5′ -ACACCATGTATTCCGGGTCAAT - 3′ for GAPDH (mouse); 5′ - GAACCTGCAGTTTGCTGTGG - 3′ and 5′ - AGAAGCGTTTGCGGTACTCA -3′ for COX-2 (mouse).

### 2.8 Lung histopathology

Lung tissues were fixed with 10% neutral formalin after mice had been sacrificed. Fixed sections were embedded in paraffin, sectioned and stained with H&E to examine infiltration of inflammatory cells. Staining [Alcian blue and periodic acid- Schiff (AB-PAS), Masson] was used to evaluate mucin-positive goblet cells (GCs).

### 2.9 Measurement of airway hyper-responsiveness

Within 24h of the last OVA challenge, AHR was assessed using whole-body plethysmography (Buxco 150 Electronics, Troy, NY, USA). Mice were anesthetized by i.p. injection of pentobarbital sodium at a dose of 50 mg/kg body weight (catalog no. 57-33-0, Sigma-Aldrich, St Louis, MO, USA). Mice underwent a tracheotomy and tracheal tubes were inserted. Then the mouse was put into the body plethysmograph chamber and the inserted tracheal tubes were connected to a ventilator.

#### 2.9.1 Analysis of immunoglobulin E OVA-specific IgE in serum

The animals were sacrificed 24 h after calculating AHR, and blood was gathered from the postcaval vein. The serum-containing supernatant was obtained after centrifugation at 800× *g* for 20 min. The serum levels of IgE were evaluated using an enzyme-linked immunosorbent assay (ELISA) kit (BioLegend, San Diego, CA, USA) according to the supplier’s instructions. Absorbance was detected at 450 nm using a plate reader of ELISA (Bio-Rad Laboratories, Hercules, CA, USA).

### 2.10 Analysis of BALF

Following blood collection, BALF was collected from the mice and processed as previously described (28). Briefly, after the tracheotomy, an endotracheal syringe was intubated into the trachea. After ice-cold PBS (0.7 mL) was injected into the lungs and recovering it twice (1.4 mL of total volume), BALF was centrifuged at 800×*g* for 10 min, and the supernatant was stored at -80°C for analysis of type 2 cytokines. To distinguish cell types, the pellet was resuspended and the suspended cells were attached to the slide using cytospin (Hanil Science Industrial, Seoul, Republic of Korea) (200× *g* at 4°C for 10 min). Cells on dried slides were fixed and stained with Diff-Quik^®^ reagent (Sysmex Co., Kobe, Japan). The levels of the type 2 cytokines interleukin-4, -13, and -17 were determined via a commercial enzyme-linked immunosorbent assay (ELISA, RayBiothech Life, Incorporation, Georgia Norcross) and detected at 450 nm using a plate reader of ELISA (Bio-Rad Laboratories).

### 2.11 Measurement of the thickness of smooth muscle and the airway wall

We examined isolated intact small bronchioles by light microscopy to measure the thickness of the airway wall and smooth muscle layer. Image-Pro Plus version 6.0 was used to determine the internal smooth muscle area (Wam1), external smooth muscle area (Wam2), bronchial luminal area (Wat1), total bronchial wall area (Wat2), and basement membrane perimeter (Pbm) in the bronchioles. The thicknesses of the smooth muscle layer (29) and airway wall (Wan) were calculated as follows:

Wan =(Wat1 – Wat2)/Pbm
Wat =(Wam1 – Wam2)/Pbm

### 2.12 Histopathological analysis of the lung tissue

At 48 h after the last OVA challenge, mice were sacrificed for histological examination. Lung tissue was removed and fixed in 10%(v/v) neutral-buffered formalin then dehydrated, embedded in paraffin, and cut into 4 μm sections that were deparaffinized with xylene and then stained with hematoxylin and eosin (H&E) and periodic acid Schiff (PAS) (both from Sigma-Aldrich). Stained sections were analyzed under a light microscope (Axio Imager M1; Carl Zeiss, Oberkochen, Germany). The degree of lung inflammation and goblet cell hyperplasia was scored on a subjective scale of 0 to 4 as previously described (30). Briefly, to score the inflammatory cell infiltration in the intraluminal, alveolar, peribronchial, and perivascular regions, cell counts were performed blind based on five point grading system for the following features: 0: normal, 1: few cells, 2: a ring of inflammatory cells 1 cell layer deep; 3: a ring of inflammatory cells 2–4 cells deep, 4: a ring of inflammatory cells of > 4 cells deep. For the quantification of goblet cells in the bronchi and bronchioles, five point grading system was used, 0: < 0.5% PAS positive cells, 1: < 25%, 2: 25-50%, 3: 50-75% and 4: > 75%. Five fields were counted for each slide and mean score was calculated from five animals. Quantification of the PAS-positive goblet cells was expressed as the number of PAS-positive cells per mm of basement membrane to correct for airway size (20).

### 2.13 Culture of cells

The human bronchial epithelial cell line Beas-2B was obtained from Shanghai Institutes for Biological Sciences (Shanghai, China). Cells were cultured in Dulbecco’s modified Eagle’s medium (Hyclone, Julich, Germany) supplemented with 10% fetal bovine serum (Gibco, Grand Island, NY, United States). All cells were grown at 37 °C inhumidified air in an atmosphere of 5% CO_2_.Cells were treated with the indicated concentration of 1,8-cineol according to experimental requirements; the same volume of DMEM was used as the solvent control.

### 2.14 Western blotting

Lung tissues were minced and homogenized by RIPA lysate containing protease and phosphatase inhibitor (Sangong Biotech,C500019-0001), followed by 12,000g centrifugation for 10 min at 4°C, and the supernatants of each group were collected. The concentration of total protein was quantified by Pierce BCA Protein Assay Kit (Thermo Scientific). Electrophoresis separation was performed by 10% SDS-PAGE and transferred with 0.25 μm PVDF membranes. The membranes were blocked with 5% milk at room temperature for 1 h, then incubated with primary antibodies (NF-κB p-P65、NF-κB P65、COX-2、Cell Signaling Technology, Dilution: 1:1000) at 4°C over- night, followed by HRP-conjugated secondary antibody (Dilution: 1:1000 or 1:10000) incubation at room temperature for 1 h. Images were obtained by ultimate exposure using LAS-4000 mini visualizer (Fujifilm Corporation, Tokyo, Jap).

### 2.15 Statistical analysis

SPSS20.0 was used for statistical analysis. Data were expressed as mean SD. The statistical significance of differences between more than two groups was calculated using one-way analysis of variance (ANOVA). Pairwise comparisons were conducted using the LSD method. All data are presented in the form of mean ± standard deviation (Mean ± SD).Data were analyzed and graphs were prepared using GraphPad Prism 9 software (GraphPad Software, San Diego, CA, USA). P < 0.05 was accepted as statistically significant.

## 3 Results

### 3.1 Effect of 1,8-cineol on OVA-induced airway hyper-responsiveness

**Figure 2:**
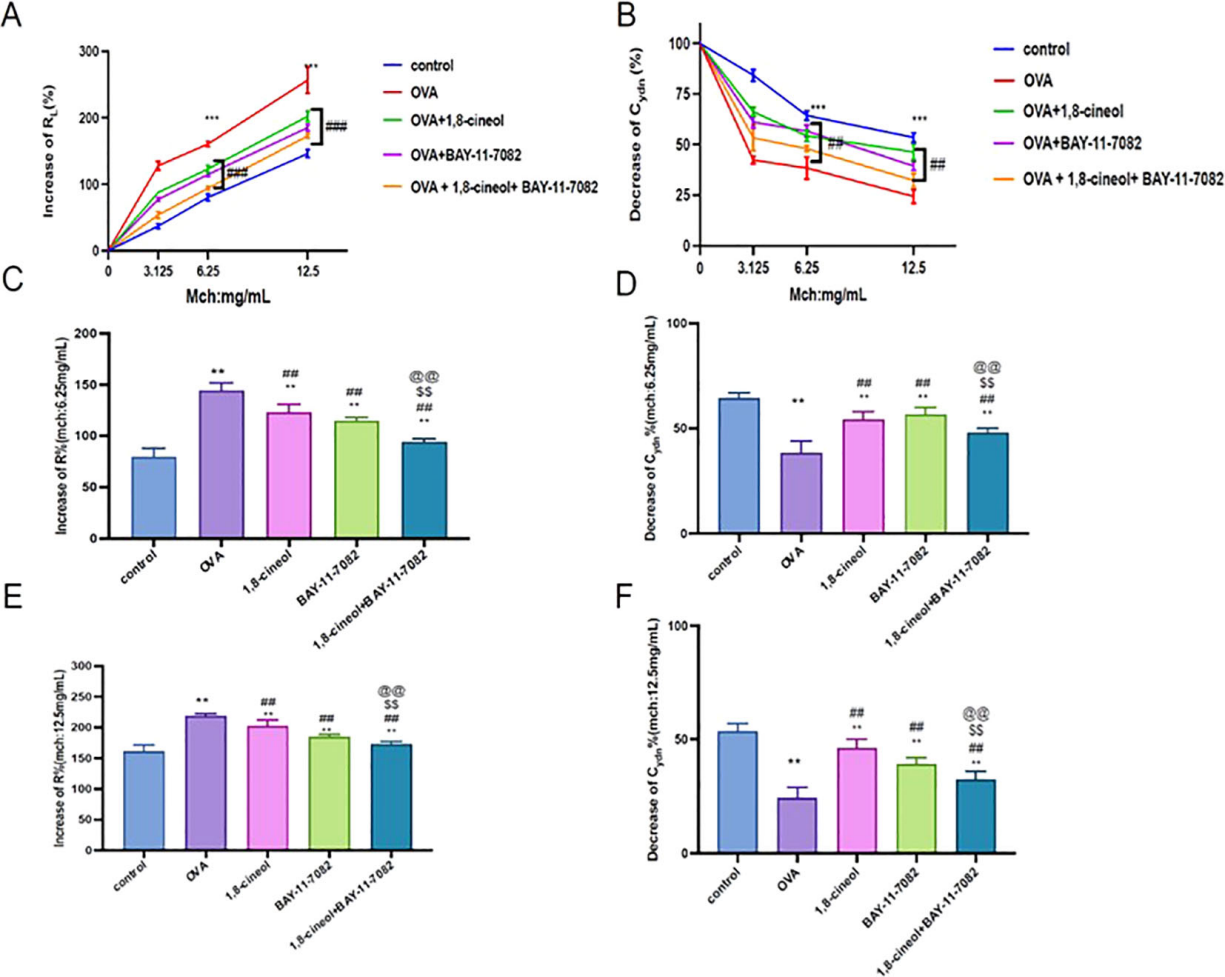
The effects of 1,8-cineol on Lung function of mice in each group. **(A, B)** Airway resistance and dynamic compliance of mice with different concentrations of methacholine; **(C, D)** Airway resistance and dynamic compliance of mice in each group at concentrations of 6.25 mg/mL methacholine; Data are presented as the mean ± standard deviation and one-way ANOVA was applied to detect significant differences; **(E, F)** Airway resistance and dynamic compliance of mice in each group at concentrations of 12.5 mg/mL methacholine; Data are presented as the medians (P25, P75) and Kruskal-Wallis ANOVA was used for comparison. N = 6 in each group. **p<0.01 vs Control, ***p<0.001 vs OVA, ^##^p<0.01 vs OVA, ^$$^p<0.01 vs OVA+eucalyptol, @@p<0.01 vs OVA + BAY-11-7082. R, airway resistance; Cdyn, dynamic compliance; Con, control group; OVA, asthma group; 1,8-cineol: OVA + 1,8-cineol group (50mg/kg);BAY-11-7082:BAY-11-7082(20mg/kg);1,8-cineol+BAY-11-7082:OVA+BAY-11-7082 (20mg/kg) + 1,8-cineol (50mg/kg).

Allergic asthma is characterized by AHR, which is evidenced by airway resistance increase (R_L_) and lung dynamic compliance decrease (C_dyn_) (31). In present study, we observed these two indicators in each group (**Figure 2**). Compared with the control group, an increase in airway resistance accompanied by a decrease in C_dyn_ were observed in the asthma groups by the increase of methacholine concentration, suggesting airways were hyper-responsive. At the concentration of 6.25 mg/mL and 12.5 mg/mL methacholine, The airway resistance in the 1,8-cineol and BAY-11–7082 group were significantly reduced. C_dyn_ was significantly increased in the 1,8-cineol and BAY-11–7082 group at 6.25 mg/mL and 12.5 mg/mL.

### 3.2 1,8-cineol reduces the thickness of smooth muscle and the airway wall

**Figure 3:**
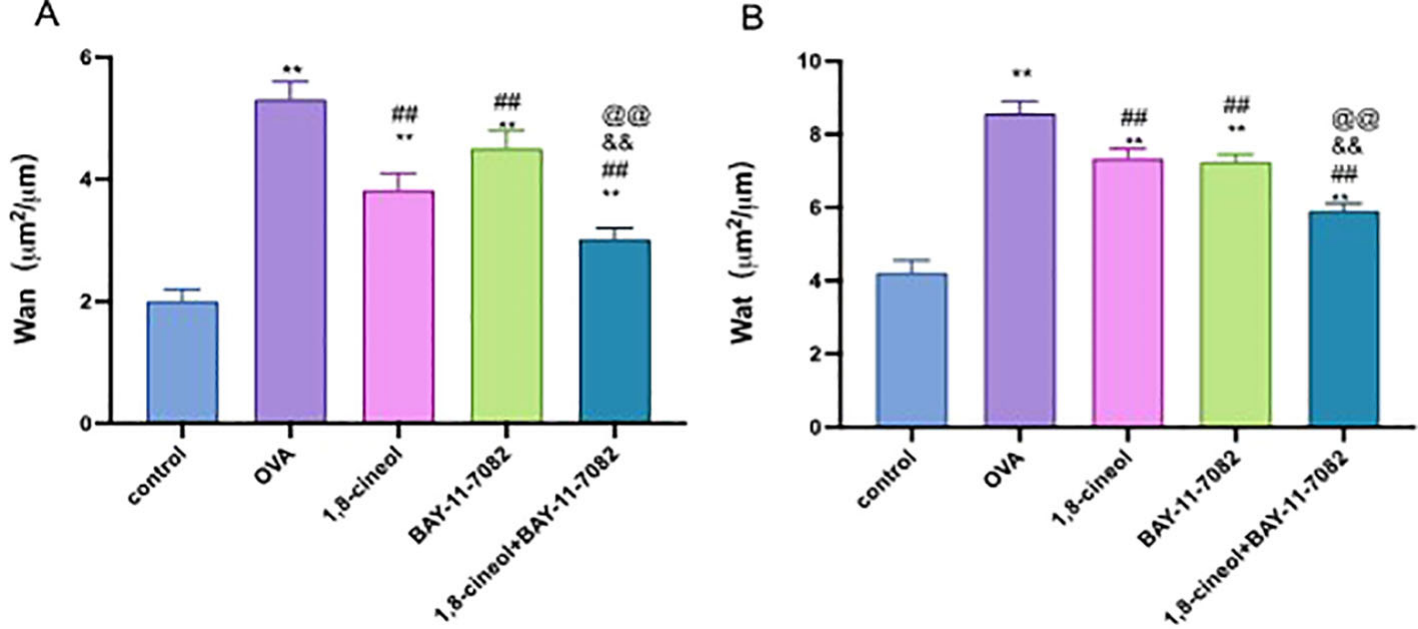
1,8-cineol reduces the thickness of airway smooth muscle and the airway wall in intact small bronchioles of asthmatic rats. Values are means ± SD (n=6); p** p<0.01 vs Control, p^##^p<0.01 vs OVA, p^&&^p<0.01 vs OVA + eucalyptol, p ^@@^p<0.01 vs OVA + BAY-11-7082.

Isolated intact small bronchioles of 1,8-cineol-treated and untreated asthmatic mice are shown in **Figure 3**. Thickness of the airway wall and smooth muscle layer was enhanced in the lung tissue of asthma group than control group of mice. However, treatment with 1,8-cineol significantly (p< 0.01) reduces the thickness of both (airway wall and smooth muscle) in the lung tissue of asthmatic mice.

### 3.3 Pathological changes in the lungs of mice

**Figure 4:**
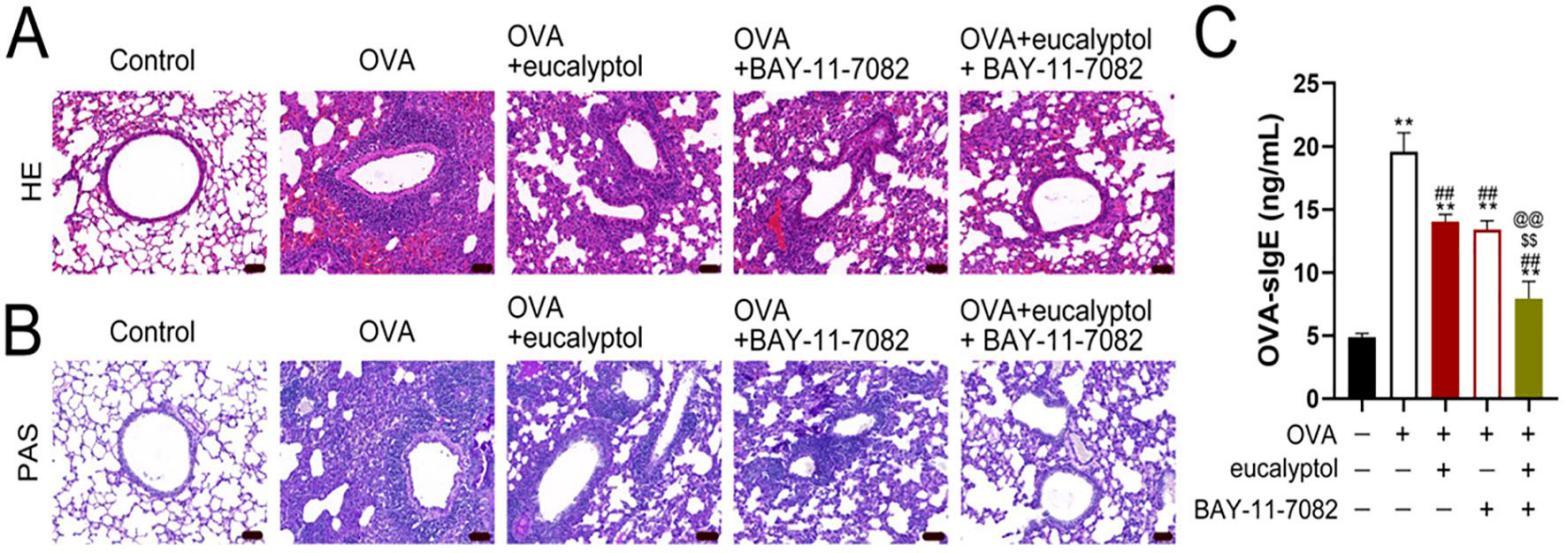
1,8-cineol treats asthma through the NF-Kb/COX-2 signaling pathway. [**(A)** HE staining; **(B)** PAS staining; **(C)** ELISA detection. Values are means ± SD (n=6); p**p<0.01 vs Control, p^##^p<0.01 vs OVA, p^$$^p<0.01 vs OVA +eucalyptol, p^@@^p<0.01 vs OVA + BAY-11-7082.].

HE staining results are shown in the **Figure 4A**. In the Control group, the bronchial and lung tissue structure was normal, there were no inflammatory cells in the bronchial tube lumen, and the tube wall thickness and results were normal. In OVA group, various inflammatory cells such as eosinophils and lymphocytes were infiltrated and aggregated around the airway. Pathological changes such as bronchial smooth muscle spasm, lumen stenosis and tube wall thickening were observed. The airway of OVA + eucalyptol and OVA + BAY-11–7082 mice had a small amount of inflammatory cells infiltrating and slightly thickened, but to a lesser extent than that of OVA + eucalyptol and OVA + BAY-11-7082. Inflammatory cells were still infiltrated around the airway of OVA + eucalyptol + BAY-11–7082 mice, but significantly reduced compared to OVA + eucalyptol + Bay-11-7082. The pathological changes such as tracheal smooth muscle spasm and lumen stenosis were improved to varying degrees. The general trend of damage degree of HE staining was: Control< “OVA + eucalyptol + BAY-11-7082”< OVA + Eucalyptus, OVA + BAY-11-7082<OVA.

The results of PAS staining are shown in the **Figure 4B**. In the Control group, there were very few goblet cells in the trachea and bronchus, and only a small amount of purplish red was expressed around the bronchus. In the OVA group, a large number of goblet cell hyperplasia was observed in the airway of mice, and the purplish red area around the bronchus was significantly increased. In “OVA + eucalyptol” and “OVA + BAY-11-7082” group, the cell hyperplasia was reduced, and the positive area around the bronchus was reduced to varying degrees. In “OVA + eucalyptol + BAY-11-7082” group, the cell proliferation was further alleviated and the positive area around the bronchus was further reduced. The general trend of PAS stained goblet cell proliferation was Control < OVA + eucalyptol + BAY-11-7082 < OVA + eucalyptol, OVA + BAY-11-7082 < OVA.

### 3.4 Effects of 1,8-cineol on the levels of IgE in serum from OVA-induced asthmatic mice

The OVA specific antibody IgE content in serum was detected by ELISA (**Figure 4**). Compared with the Control group, the serum levels of OVA specific antibody IgE in the other groups were up-regulated. Serum levels of OVA specific antibody IgE in “OVA + eucalyptol + BAY-11-7082”, “OVA + eucalyptol”, “OVA + BAY-11-7082” were down-regulated but still higher than those in Control group. There was no statistical difference in serum OVA specific antibody IgE content between “OVA + eucalyptol” and “OVA + BAY-11-7082” groups. The general trend of serum OVA specific antibody IgE content detected by ELISA was: Control < “OVA + eucalyptol + BAY-11-7082” < “OVA + eucalyptol”, “OVA + BAY-11-7082” < OVA.

### 3.5 Effects of 1,8-cineol on inflammatory cell counts in BALF from asthmatic mice

**Figure 5:**
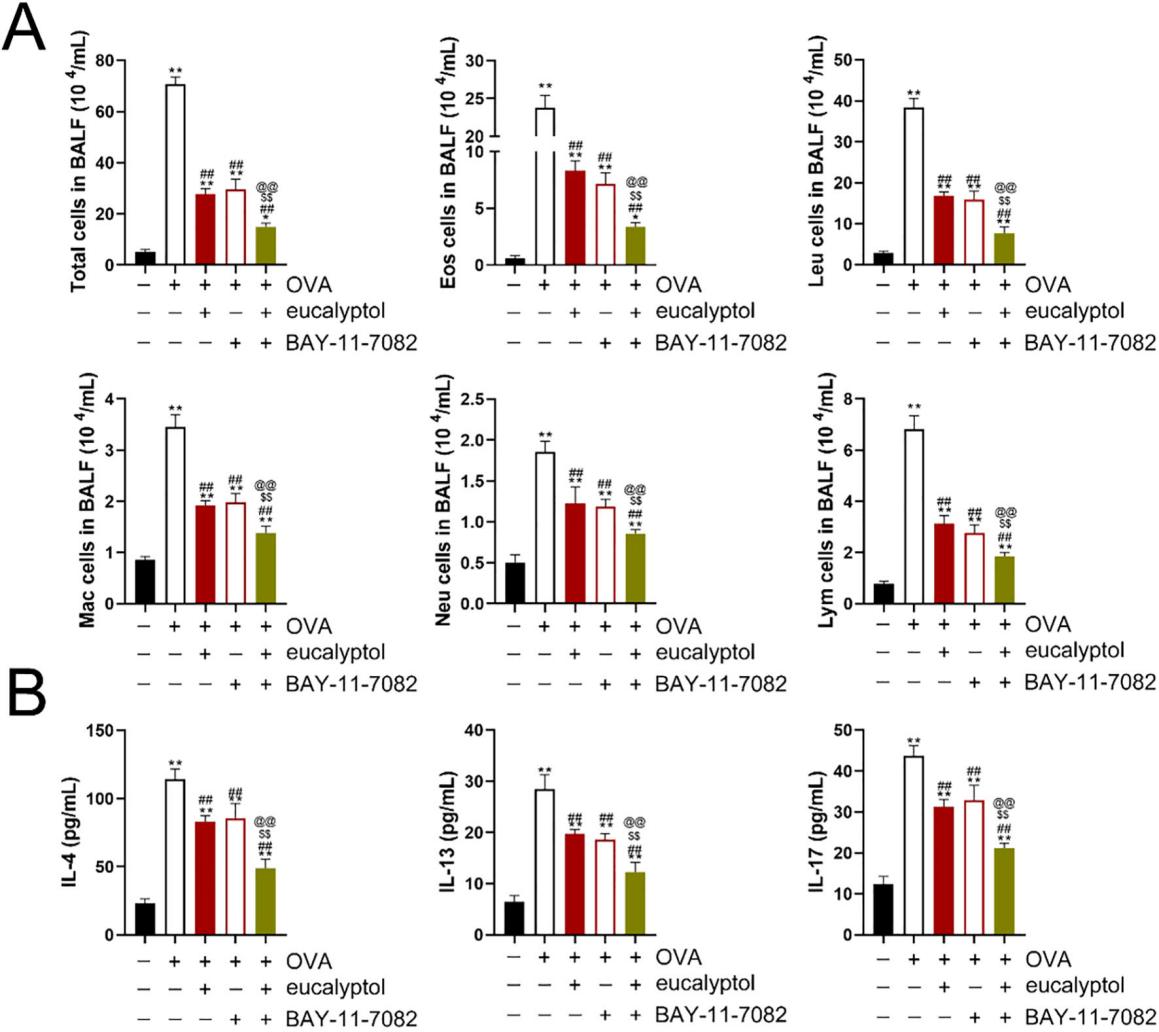
**(A)** 1,8-cineol (eucalyptol)reduced the inflammatory cell count in bronchoalveolar lavage fluid. **(B)** 1,8-cineol reduced the levels of interleukin (IL)-4, IL-13 and IL-17 in the bronchoalveolar lavage fluid. Control:mice treated with phosphate-buffered saline only; ovalbumin (OVA): mice sensitized and challenged with OVA. OVA+ eucalyptol: asthma model combined with 1,8-cineol treatment group. OVA + BAY-11-7082: asthma model combined with BAY-11-7082 treatment group. OVA + eucalyptol + BAY-11-7082: asthma model combined with BAY-11-7082 and 1,8-cineol. Values are presented as means ± SD (n=6). p**:p<0.01 vs Control, p^##^:p<0.01 vs OVA, p^$$^:p<0.01 vs OVA+eucalyptol, p^@@^:p<0.01 vs “OVA + BAY-11-7082”.

Alveolar lavage fluid (BALF) was collected and analyzed (**Figure 5**). Compared with the Control group, the number of total cells, white blood cells, eosinophils, neutrophils, lymphocytes and macrophages in the other groups were up-regulated. Compared with the OVA group, Total cells, white blood cells, eosinophils, neutrophils, lymphocytes, and macrophages in “OVA+ eucalyptol +BAY-11-7082”, “OVA + eucalyptol” and “OVA + BAY-11-7082” were downregulated but remained higher than those in Control group. There was no statistically significant difference in the number of total cells, white blood cells, eosinophils, neutrophils, lymphocytes, and macrophages on “OVA + eucalyptol” and “OVA+BAY-11-7082”. The number of white blood cells, eosinophils, neutrophils, lymphocytes and macrophages was as follows: Control<OVA + eucalyptol + BAY-11-7082”< “OVA + eucalyptol”, “OVA + BAY-11-7082”< OVA.

### 3.6 Effects of 1,8-cineol on the activity and expression of NF-κB p-P65, NF-κB P65, COX-2 in lung tissue from OVA-induced asthmatic mice

**Figure 6:**
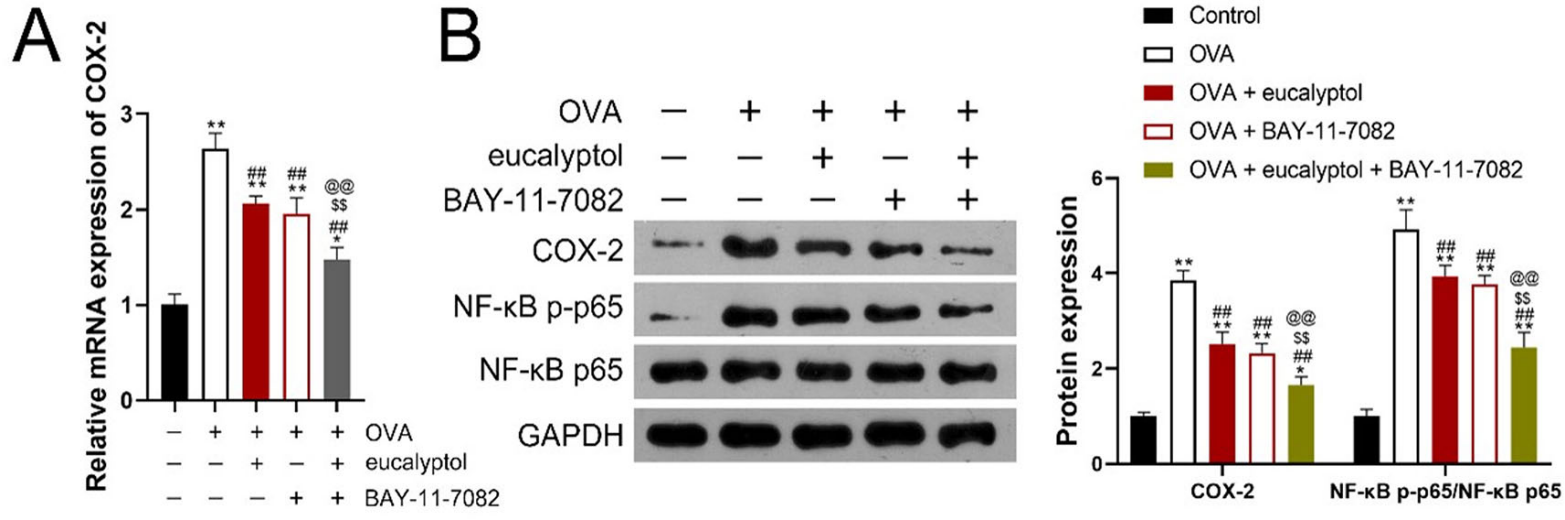
**(A)** PCR detection; **(B, C)** Western blot assay; Control:mice treated with phosphate-buffered saline only;ovalbumin(OVA): mice sensitized and challenged with OVA. OVA+ eucalyptol: asthma model combined with 1,8-cineol treatment group. OVA + BAY-11-7082: asthma model combined with BAY-11-7082 treatment group. OVA + eucalyptol + BAY-11-7082:asthma model combined with BAY-11-7082 and 1,8-cineol.Values are presented as means ± SD (n=6). p**:p<0.01 vs Control, p^##^:p<0.01 vs OVA, p^$$^:p<0.01 vs OVA + eucalyptol, p^@@^:p<0.01 vs “OVA + BAY-11-7082”.

The gene expression of COX-2 in lung tissue was detected by Qpcr (**Figure 6A**). Compared with the Control group,COX-2 gene expression in the other groups was upregulated, and compared with the OVA group, COX-2 gene expression in BALF of “OVA + eucalyptol + BAY-11-7082”, “OVA + eucalyptol”, “OVA + BAY-11-7082” was down-regulated but still higher than that of control group. There was no statistically significant difference in COX-2 gene expression between “OVA + eucalyptol” and “OVA + BAY-11-7082”. The general trend of COX-2 gene expression detected by qPCR was: Control < “OVA + eucalyptol + BAY-11-7082” < “OVA + eucalyptol”,” OVA + BAY-11-7082” < OVA.

The protein expression of NF-κB p-P65, NF-κB P65 and COX-2 in lung tissue was detected by WB (**Figures 6B, C**). Compared with the Control group, the expression of NF-κB p-P65/NF-κB P65 and COX-2 protein in the other groups was up-regulated. Protein expressions of NF-κB p-P65/NF-κB P65 and COX-2 in “OVA + eucalyptol + BAY-11-7082”, “OVA + eucalyptol”, “OVA + BAY-11-7082” were down-regulated but still higher than those in Control group. There was no statistically significant difference in the expression of NF-κB p-P65/NF-κB P65, COX-2 on “OVA + eucalyptol” and “OVA + BAY-11-7082”. The general trend of protein expression of COX-2, NF-κB p-P65/NF-κB P65 in lung tissue detected by WB was Control<“OVA + eucalyptol + BAY-11-7082” <“OVA + eucalyptol”, “OVA + BAY-11-7082”<OVA.

### 3.7 Effects of 1,8-cineol on the activity and expression of N-cadherin, NF-κB p-P65/NF-κB P65, COX-2 in cell groups

**Figure 7:**
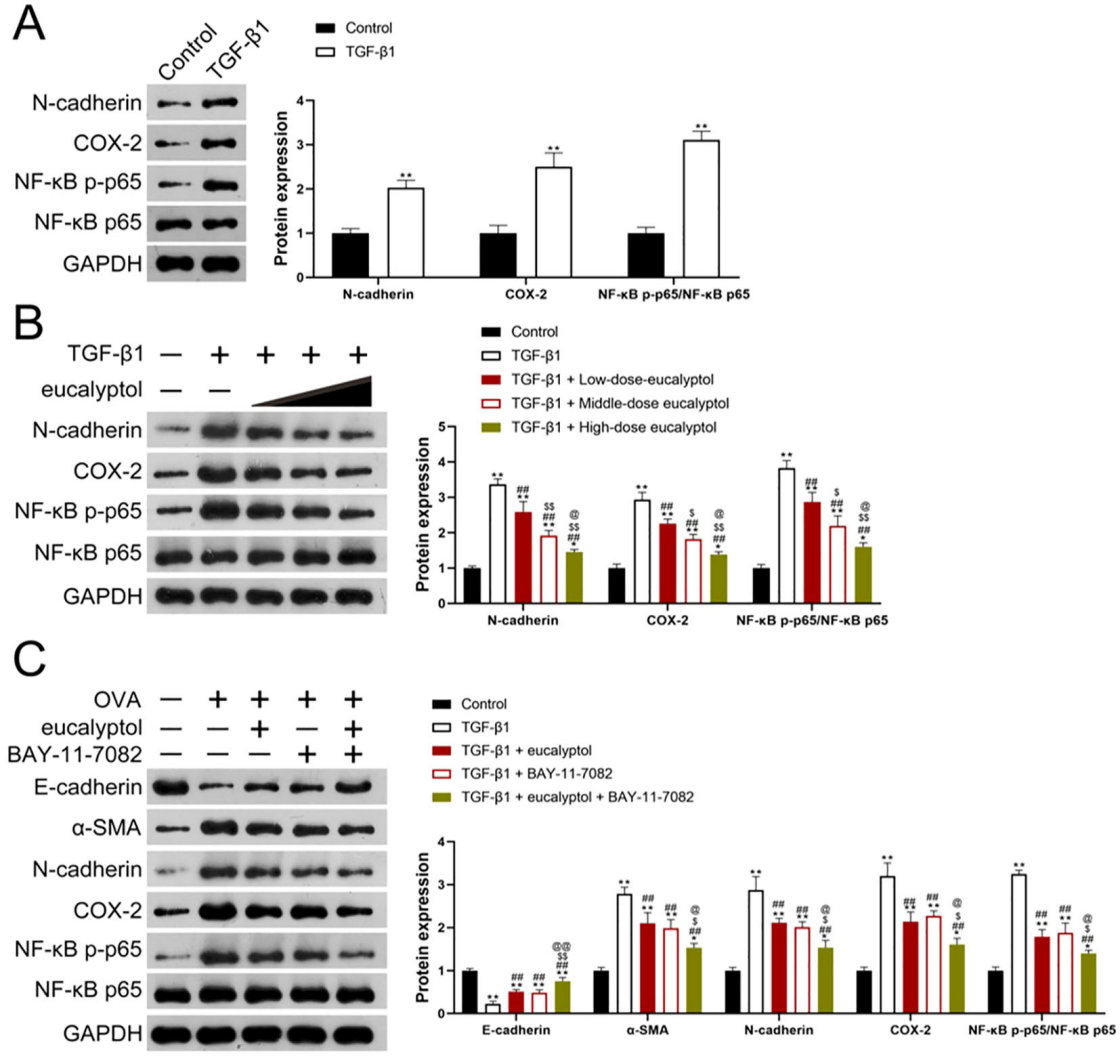
**(A)** The expressions of N-cadherin, NF-κB p-P65/NF-κB P65 and COX-2 were detected by WB. **(B)** The protein expression of N-cadherin, COX-2, NF-κB p-P65/NF-κB P65 in each group was detected by WB. **(C)** The expression of E-cadherin, α-SMA, N-cadherin, NF-κB p-P65/NF-κB P65, COX-2 in cells of each group was detected by WB. Control: mice treated with phosphate-buffered saline only; ovalbumin (OVA): mice sensitized and challenged with OVA. OVA+ eucalyptol: asthma model combined with 1,8-cineol treatment group. OVA + BAY-11-7082: asthma model combined with BAY-11-7082 treatment group .OVA + eucalyptol + BAY-11-7082: asthma model combined with BAY-11-7082 and 1,8-cineol. Values are presented as means± SD (n=6). p**:p<0.01 vs Control, p^##^:p<0.01 vs TGF-β1; p^@@^:p<0.01 vs TGF-β1 + BAY-11-7082;p$:p<0.05; p^$$^:p<0.01vs “TGF-β1+Low-dose-eucalyptol”; p@:p<0.05 vs “TGF-β1+Middle-dose eucalyptol”.

The expression of N-cadherin, NF-κB p-P65/NF-κB P65 and COX-2 in cells of each group was detected by WB (**Figure 7A**). Protein expression of N-cadherin, COX-2, NF-κB p-P65/NF-κB P65 was up-regulated in TGF-β1 group compared with Control group. The general trend of protein expression of N-cadherin, COX-2, NF-κB p-P65/NF-κB P65 detected by WB was Control<TGF-β1.

The protein expression of N-cadherin, COX-2, NF-κB p-P65/NF-κB P65 in cells of each group was detected by WB (**Figure 7B**). Protein expressions of N-cadherin, COX-2, NF-κB p-P65/NF-κB P65 in the other groups were up-regulated compared with those in the Control group. N-cadherin, COX-2, NF-κB in “TGF-β1+Low-dose-eucalyptol (5 μM)”group, “TGF-β1+mid-dose-eucalyptol l (10 μM)” group, “TGF-β1+ High-dose-eucalyptol l (20 μM)” group. The protein expression of p-P65/NF-κB P65 was down-regulated in a dose-dependent manner. The expression trend of N-cadherin, COX-2, NF-κB p-P65/NF-κB P65 in cells of all groups was TGF-β1>“TGF-β1+ Low-dose-eucalyptol”> “TGF-β1+mid-dose-eucalyptol”;”TGF-β1+High-dose eucalyptol” > Control.

### 3.8 Effects of 1,8-cineol on the activity and expression of E-cadherin, α-SMA, N-cadherin, NF-κB p-P65/NF-κB P65, COX-2 in cell groups

The expression of E-cadherin,α-SMA,N-cadherin, NF-κB p-P65/NF-κB P65 and COX-2 in cells of each group was detected by WB (**Figure 7C**). Compared with the Control group, the expression of E-cadherin protein in the other groups was down-regulated, and compared with TGF-β1 group, The expression of E-cadherin protein in “TGF-β1+ BAY-11-7082”, “TGF-β1+ eucalyptol”, and “TGF-β1+ eucalyptol + BAY-11-7082” groups was up-regulated but still lower than that in Control group. There was no statistically significant difference in the expression of E-cadherin on “TGF-β1 + eucalyptol” and “TGF-β1 + BAY-11-7082” on eucalyptol. The general trend of E-cadherin expression in cells detected by WB was as follows: TGF-β1<“TGF-β1 + eucalyptol”, “TGF-β1 + BAY-11-7082”<“TGF-β1 + eucalyptol + BAY-11-7082”<Control.

The protein expression of α-SMA, N-cadherin, COX-2, NF-κB p-P65/NF-κB P65 in the other groups were up-regulated compared with those in the Control group. The protein expressions of α-SMA, N-cadherin, COX-2, NF-κB p-P65/NF-κB in group “TGF-β1+ BAY-11-7082”,”TGF-β1+ eucalyptol” and “TGF-β1+ eucalyptol + BAY-11-7082”were down-regulated compared with those in the group of TGF-β1 and up-regulated compared with those in the control group. There was no significant difference in the protein expression of α-SMA, N-cadherin, COX-2, NF-κB p-P65/NF-κB P65 on “TGF-β1 + eucalyptol” and “TGF-β1 + BAY-11-7082”. The expression trend of α-SMA,N-cadherin,COX-2,NF-κB p-P65/NF-κB P65 detected by WB was TGF-β1>“TGF-β1 + eucalyptol”, “TGF-β1 + BAY-11-7082”>“TGF-β1 + eucalyptol + BAY-11-7082”> Control.

## 4 Discussion

Allergic asthma is associated with T helper (25) 2 cell-biased immune responses and characterized by the airway hyperresponsiveness (AHR) (32). Bronchial inflammation, smooth muscle spasm, and mucus production in allergic asthma are triggered by IL-4, IL-5,and IL-13, which are released by Th2 cells. IL-13 plays the main role in the excessive secretion of mucus and AHR. IL-5 participates in the activation and migration of eosinophils to airways triggering bronchial inflammation. IL-4 induces IgE isotype switching in B cells and upregulates high-affinity IgE receptor (FcϵRI) on the surface of target cells. Mast cells are activated upon allergen- induced cross-linking of FcϵRI-bound IgE on their plasma membrane surface. Subsequently, mast cells release histamine and other mediators that lead to allergic symptoms. The levels of IL-4, IL-5, and IL-13 are increased in the bronchoalveolar lavage (BAL) of asthmatic patients (23). The purpose of the present study was to elucidate, using an OVA-induced asthmatic mice model, the anti-asthmatic efficacy of 1,8-cineol,the major constituent of eucalyptus species, is well known for its anti-inflammatory, antioxidant, bronchodilatory, antiviral and antimicrobial effects (33). The monoterpene 1,8-cineole (eucalyptol) is chemically a terpenoid oxide that is well known as the major constituent (77–84%) of various eucalyptus species and also the component of other essential oils with a relevant meaning for clinical effect. Eucalyptus oil is well known for its biological activities, including anti-inflammatory, antioxidant, free radical scavenging, mucolytic/secretolytic, bronchodilatory, antiviral and antimicrobial effects, as reviewed elsewhere (34). The present 1,8-cineol treatment effectively decreased airway hyper-responsiveness (AHR) with a reduction in the migration of inflammatory cells and type 2 pro-inflammatory cytokine levels in BALF and IgE levels in serum, which had been elevated by OVA sensitization. These changes were consistent with the results of decreased inflammatory cell migration and decreased hyper mucinogenesis in the lung tissues of 1,8-cineol-treated mice. Additionally,1,8-cineol notably reduced the expression of COX-2,α-SMA,N-cadherin and NF-κB phosphorylation followed by reduced E-cadherin expression levels elevated by OVA exposure.

The reason why allergic asthma exhibits symptoms of asthma is due to its pathological BHR, increased inflammatory cells, and increased mucus. The pathophysiology of type II asthma is an increase in the concentration of IL-4, IL-5, and IL-13, which leads to the accumulation of type 2 related cells (such as mast cells and eosinophils) in lung tissue and increases the production of mucus. From mild allergic early-onset asthma to severe delayed onset asthma with high and ultra-high Th2, there are four different degrees of asthma phenotypes. Other inflammatory cytokines, chemokines, and growth factors are also easily induced by these events, leading to high production and collectively contributing to representative symptoms of asthma. The pathogenesis of type 2 low internal asthma is more complex, and no clear biomarkers are associated with its onset. Currently, all asthma patients without type 2 high inflammation are classified as type 2 low asthma. In this study, compared to the experimental data of the OVA group, the 1,8-cineol group showed a decrease in the degree of AHR, as well as a significant reduction in airway inflammation and lung tissue mucus production. The migration of inflammatory cells, production of type 2 cytokines in BALF, and increased serum IgE content were observed in the 1,8-cineol group. In BALF, the 1,8-cineol group showed greater inhibition of the migration of inflammatory cells, especially eosinophils, while the OVA group exhibited a dose-response relationship. In addition, the 1,8-cineol group exhibited low production of IL-4, IL-5, and IL-13, as well as low production of serum total IgE and OVA-specific IgE. Based on the above data, we infer that the protective effect of 1,8-cineol on asthma response is mainly achieved by reducing the level of type 2 cytokines.

In asthma, the NF-κB/COX-2 pathway and the TGF-β1 signal jointly promote epithelial-mesenchymal transition (EMT) through a synergistic effect, driving airway remodeling. Inflammatory stimuli (such as ovalbumin OVA) activate NF-κB, leading to the upregulation of pro-inflammatory factors (such as IL-4, IL-13) and COX-2 expression. COX-2 metabolizes arachidonic acid to produce prostaglandins, further amplifying the inflammatory response and possibly directly or indirectly enhancing the activity of TGF-β1 (23). The activation of NF-κB can induce the expression of TGF-β1. TGF-β1 promotes EMT through Smad and non-Smad pathways (such as MAPK), causing airway epithelial cells to lose polarity and acquire an interstitial phenotype (such as expressing α-SMA, N-cadherin). Moreover, TGF-β1 can also feedback activate NF-κB, forming a positive feedback loop. The NF-κB and TGF-β1 pathways share downstream effector molecules (such as Smad3), jointly promoting the transcription of EMT-related genes (35).

As a common transcription factor, NF-kB plays a role in the pathogenesis of inflammation by regulating gene expression of iNOS, COX-2, and various cytokines (35). Asthma is a disease characterized by chronic inflammation of the airways. NF-kB has the activity of promoting asthma inflammation in human and animal lung tissues, and its role in the pathogenesis of asthma has been fully demonstrated in many clinical trials and various animal experiments (36, 37). In addition, there are studies involving NF-B-involved cascades, confirming that iNOS and COX-2 are present in NF- kB phosphorylation downstream plays a role (38). COX-2 is a protein that plays a major role in the inflammatory response, and an increase in its level can induce the production of prostaglandins, leading to the inflammatory response (39). The protective effect of natural products on asthma models by regulating the expression of COX-2 has been fully confirmed. Transcription factors such as NF-κB regulate the expression of hundreds of genes involved in critical cellular processes, including cell growth, differentiation, inflammation, development, and apoptosis. Many inflammatory target proteins-such as matrix metalloproteinase-9 (MMP-9), intercellular adhesion molecule-1 (ICAM-1), vascular cell adhesion molecule-1 (VCAM-1), cyclooxygenase-2 (COX-2), and cytosolic phospholipase A2 (cPLA2)-are key components of inflammatory signaling pathways activated by diverse stimuli. Among these, cyclooxygenases and lipoxygenases are pivotal enzymes that catalyze the metabolism of arachidonic acid into prostaglandins and leukotrienes, both of which play central roles in inflammatory responses. In the present study, intranasal 1,8-cineol significantly suppressed OVA-induced COX-2 expression in a mouse model of chronic asthma. This effect may be linked to the inhibition of OVA-induced nuclear translocation of NF-κB, as demonstrated by 1,8-cineol treatment, which similarly correlates with reduced COX-2 expression. In conclusion, our results suggested that 1,8-cineol can effectively improve lung function, alleviate airway inflammation, reduce airway mucus secretion and collagen deposition in allergic asthmatic mice.

## 5 Limitation

The study is limited to one cell line and one murine model. validation in primary human bronchial epithelial cells or alternative asthma models would strengthen translational relevance. While the NF-κB/COX-2 axis is well demonstrated, the involvement of upstream kinases (e.g., IKKα/β) or downstream inflammatory mediators could be briefly discussed.

## 6 Conclusion

**Figure 8:**
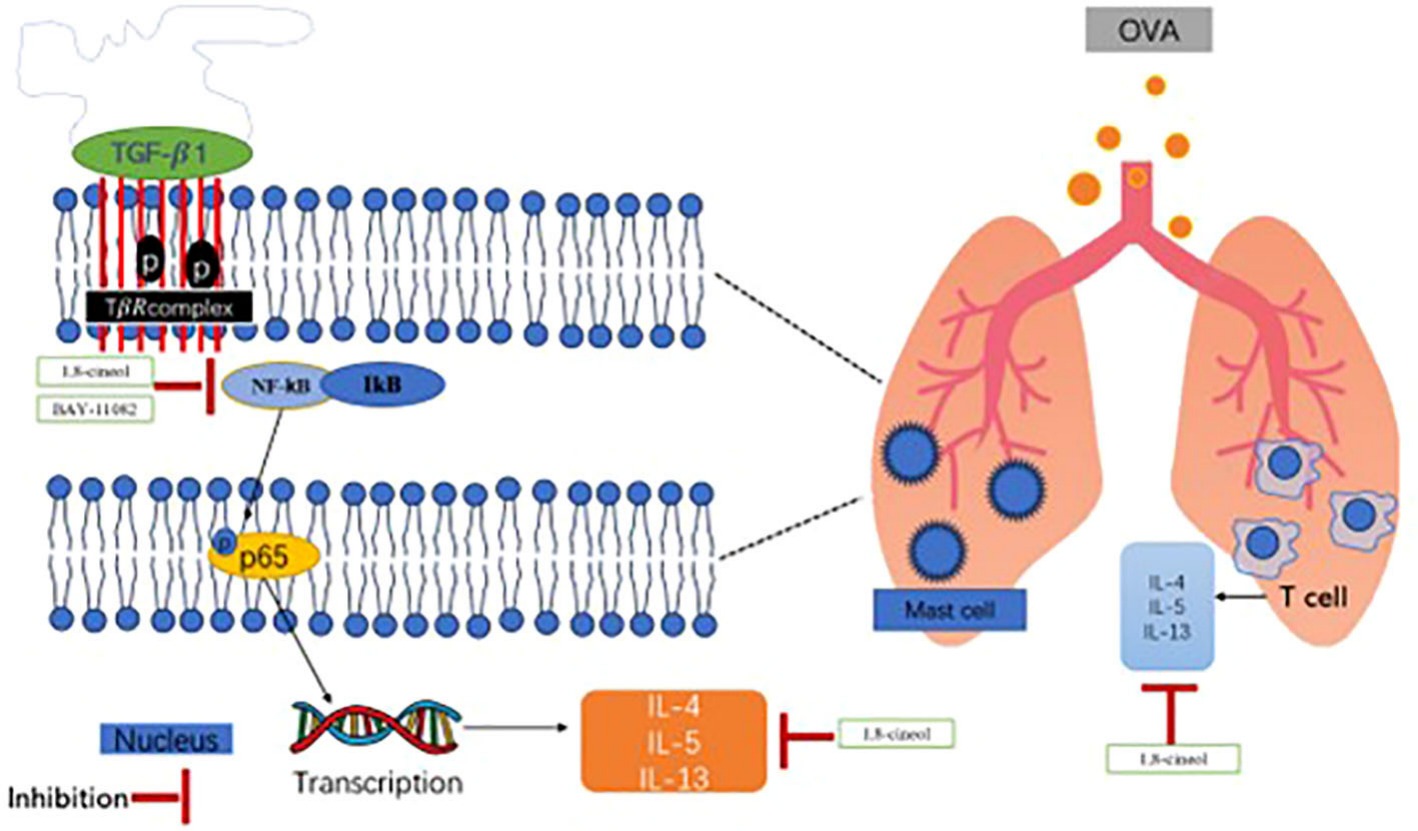
1,8-cineol inhibits macrophage activation in an experimental model of asthma. NF-κB/p-P65 exerts a significant role in TGF-β1-induced macrophage activation. The biological activities of macrophage include cell proliferation, cell migration. 1,8-cineol can effectively inhibit TGF-β1- mediated macrophage activation.

We demonstrated that 1,8-cineol inhibited airway inflammation and macrophage activation in a mouse model of OVA-induced asthma. 1,8-cineol could suppress TGF-β1-induced macrophage activation, including cell proliferation, cell migration. The inhibitive effects of 1,8-cineol in an experimental model of asthma may be mediated by the NF-κB/p-P65 signaling pathway (**Figure 8**). Our findings support the potential application of 1,8-cineol as a therapeutic drug against allergic asthma.

This paper was previously posted on Research Square under DOI: 10.21203/rs.3.rs-4880907/v1 (public release date: September 9, 2024).

## Data Availability

The original contributions presented in the study are included in the article/**Supplementary Material**. Further inquiries can be directed to the corresponding authors.
