# Supplementary material for: Anti-inflammatory effects of 1,8-cineol via NF-κB/COX-2 pathway in BEAS-2B cells and alleviates bronchoconstriction and airway hyperreactivity in ovalbumin sensitized mice
Source: Front Immunol. 2026 Mar 27;17:1714915. doi: 10.3389/fimmu.2026.1714915 (PMC13065646; doi:10.3389/fimmu.2026.1714915)
Supplement: Supplementary file 1 [file DataSheet1.pdf]

Supplementary Material

Supplementary Figure 1. Uncropped and unprocessed blots for Figure 6.

Supplementary Figure 2. Uncropped and unprocessed blots for Figure 7.

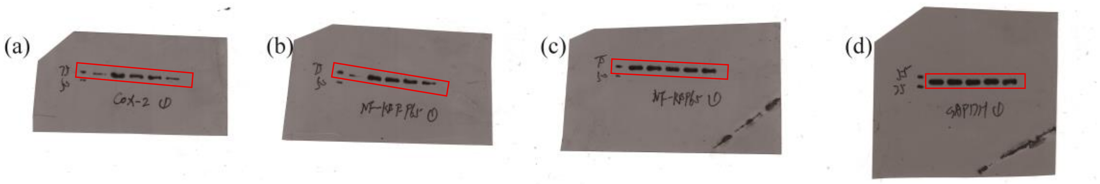

Supplementary Figure 1. Uncropped and unprocessed blots for Figure 6. Full, uncropped scans of Western blots used for the quantification of protein expression in lung tissue samples from the different experimental groups in Figure 6. **(a)** COX-2. **(b)** Phosphorylated NF-κB p65 (p-P65). **(c)** Total NF-κB p65. **(d)** GAPDH (Loading Control). The red boxes indicate the cropped regions presented in the main Figure 6B. Molecular weight markers (in kDa) are shown on the left of each blot. The lanes are loaded as follows: Lane 1: Control; Lane 2: OVA; Lane 3: OVA + 1,8-cineol; Lane 4: OVA + BAY-11-7082; Lane 5: OVA + BAY-11-7082 + 1,8-cineol

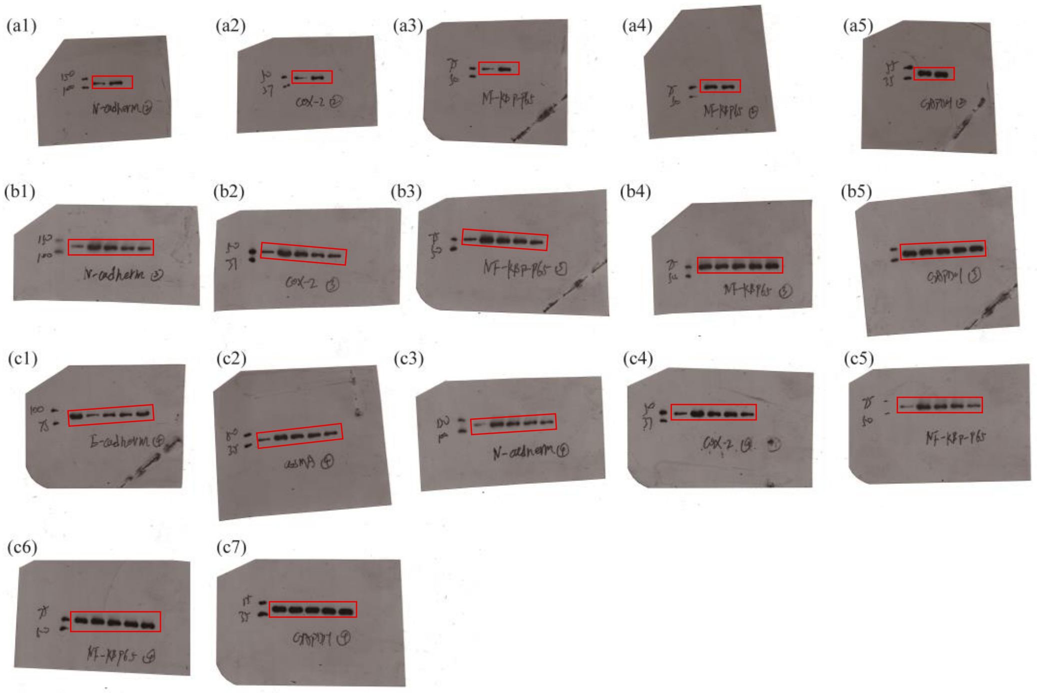

Supplementary Figure 2. Uncropped and unprocessed blots for Figure 7. Full, uncropped scans of Western blots used for the quantification of protein expression in TGF-β1-stimulated BEAS-2B cells. The red boxes indicate the cropped regions presented in the main Figures 7A, 7B, and 7C. Molecular weight markers (in kDa) are shown on the left of each blot.

(a1-5) Blots corresponding to Figure 7A. Lanes are loaded as follows: 1. Control; 2. TGF-β1.

Blots for N-cadherin (a1), COX-2 (a2), NF-κB p-P65 (a3), NF-κB p65 (a4) and GAPDH (a5) are presented.

(b1-5) Blots corresponding to Figure 7B (Dose-dependent effects). Lanes are loaded as follows: 1. Control; 2. TGF-β1; 3. TGF-β1 + Low-dose 1,8-cineol (5 μM); 4. TGF-β1 + Mid-dose 1,8-cineol (10 μM); 5. TGF-β1 + High-dose 1,8-cineol (20 μM).

Blots for N-cadherin (b1), COX-2 (b2), NF-κB p-P65 (b3), NF-κB p65 (b4) and GAPDH (b5) are presented.

(c1-7) Blots corresponding to Figure 7C (Inhibitor effects). Lanes are loaded as follows: 1. Control; 2. TGF-β1; 3. TGF-β1 + 1,8-cineol; 4. TGF-β1 + BAY-11-7082; 5. TGF-β1 + 1,8-cineol + BAY-11-7082.

Blots for E-cadherin (c1), α-SMA (c2), N-cadherin (c3), COX-2 (c4), NF-κB p-P65 (c5), NF-κB p65 (c6) and GAPDH (c7) are presented.
